# The development of rapidly progressive glomerulonephritis associated with both antineutrophil cytoplasmic antibody-associated vasculitis and anti-glomerular basement membrane nephritis in the course of nontuberculous mycobacterium infection: a case report

**DOI:** 10.1186/s41927-020-00167-y

**Published:** 2020-12-14

**Authors:** Mikiya Kato, Risa Wakiya, Tomohiro Kameda, Kousuke Inoue, Tadashi Sofue, Yusuke Ushio, Koichi Sugihara, Shusaku Nakashima, Hiromi Shimada, Mai Mahmoud Fahmy Mansour, Norimitsu Kadowaki, Hiroaki Dobashi

**Affiliations:** 1 Department of Internal Medicine, Division of Hematology, Rheumatology and Respiratory Medicine, Faculty of Medicine, Kagawa University, 1750-1 Ikenobe, Miki-cho, Kita-gun, Kagawa, 761-0793 Japan; 2 Department of Diagnostic Pathology, Faculty of Medicine, Kagawa University, 1750-1 Ikenobe, Miki-cho, Kita-gun, Kagawa, 761-0793 Japan; 3 Department of CardioRenal and CerebroVascular Medicine, Faculty of Medicine, Kagawa University, 1750-1 Ikenobe, Miki-cho, Kita-gun, Kagawa, 761-0793 Japan

**Keywords:** Nontuberculous mycobacteriosis, Myeloperoxidase-antineutrophil cytoplasmic antibody, Anti-glomerular basement membrane antibody, Rapidly progressive glomerulonephritis

## Abstract

**Background:**

Antineutrophil cytoplasmic antibodies (ANCA) and Anti-glomerular basement membrane (GBM) antibodies often induce rapidly progressive glomerulonephritis (RPGN). Some reports have demonstrated RPGN with the sequential appearance of ANCA then anti-GBM antibodies, suggesting that ANCA may induce the development of anti-GBM antibodies. Whereas, many reports have shown that the development of ANCA is associated with various infectious diseases, such as non-tuberculous mycobacterial infection.

**Case presentation:**

A 65-year-old woman with pulmonary non-tuberculous mycobacterial (NTM) infection was monitored without treatment. One year later, serum myeloperoxidase (MPO)- ANCA were elevated (14.1 U/mL (normal value < 3.0 U/ml)). A high fever and RPGN appeared 1 year later, and serum MPO-ANCAs were 94.1 U/mL. Anti-GBM antibodies were also detected. A renal biopsy revealed crescentic glomerulonephritis with linear deposits of IgG and C3c along the GBM and interstitial inflammation with endarteritis of arterioles. The diagnosis was RPGN associated with anti-GBM nephritis and ANCA-associated vasculitis.

**Conclusion:**

This report shows that preceding NTM infection may have induced ANCA and anti-GBM antibodies and caused the development of RPGN.

## Background

Anti-glomerular basement membrane (GBM) disease is often used synonymously with “Goodpasture’s disease” or “Goodpasture syndrome”. This disorder is caused by circulating autoantibodies associated with the intrinsic antigen located inside the GBM. Rapidly progressive glomerulonephritis (RPGN) and pulmonary hemorrhage are common manifestations in this disorder. The antigen targeted by anti-GBM antibodies is the NC1 domain of the α3 chain of type IV collagen [1]. The normal structural configuration of type IV collagen hexamers in the GBM could prevent antigen-antibody interactions. Some reports have demonstrated RPGN with the sequential appearance of antineutrophil cytoplasmic antibodies (ANCA) then anti-GBM antibodies [2, 3].

ANCA-associated vasculitis (AAV) is classified into granulomatosis with polyangiitis (GPA), microscopic polyangiitis (MPA), and eosinophilic granulomatosis with polyangiitis (EGPA), and is defined as small-vessel vasculitides. Many reports have shown that the development of ANCA is associated with various infectious diseases [4]. Some case reports have focused on the development of AAV secondary to pulmonary nontuberculous mycobacterial (NTM) infections caused predominantly by *M. avium* or M. intracellulare (belonging to *Mycobacterium avium* complex; MAC) [5, 6]. This is a case study of RPGN that was preceded by NTM infection, followed by an ANCA response and then later by an anti-GBM antibody response. Both ANCA and anti-GBM antibodies are autoantibodies that could induce RPGN. It is possible, therefore, that NTM-induced ANCA, followed by the subsequent production of anti-GBM antibodies can lead to RPGN.

## Case presentation

A 65-year-old woman was admitted to our hospital with complaints of fever and general malaise enduring the previous week. She is an ex-smoker with 5 pack years smoking history from 20 to 30 years old, although she had quit smoking at 30 years old. She had never had any other inhalant exposure. She had been diagnosed with polyarteritis nodosa (PAN) 3 years prior. The clinical symptoms at the time of PAN onset were fever and myalgia, but no other clinical symptoms were observed. At that time, her serum ANCA was negative. We had carefully discussed with some pathologists for the possibility of an ANCA-negative AAV at the time of diagnosis of PAN. At the time of PAN diagnosis, CT imaging revealed a microaneurysm, which was often revealed in PAN, in the renal artery. Histological findings of the biceps muscle biopsy, which showed muscle damage by MRI, showed necrotizing vasculitis of the medium-sized artery without small or capillary lesions. Furthermore, there were no abnormalities in the urine findings, which are often seen in MPA, nor were there any findings suggesting glomerular lesions. Based on the above findings, the patient was diagnosed with PAN, a necrotizing vasculitis confined to medium-sized arteries. At that time, the patient was given remission induction therapy in the form of prednisolone (PSL) 1 mg/kg/day combined with cyclophosphamide pulse (15 mg/kg/day), and the patient improved. Maintenance therapy of steroids alone was then administered, and the dosage of prednisolone was reduced to 3 mg/day about 9 months prior to the current admission. No renal lesions or ANCA had been observed since the PAN diagnosis. At the time of diagnosis of PAN, the patient had a pulmonary lesion on CT and was monitored by a respiratory specialist, but the diagnosis of NTM had not yet been made. Subsequently, the CT findings of the lung lesions also showed deterioration, and sputum cultures showed positive findings of NTM. The respiratory specialist had carefully considered the timing of therapeutic intervention for NTMs in view of the interaction with PAN medications. One year after the diagnosis of pulmonary NTM infection, the patient tested positive for serum myeloperoxidase (MPO)-ANCA (14.1 U/mL (normal value < 3.0 U/mL)); however, because there were no systemic manifestations, the patient was monitored without treatment.

**Fig. 1:**
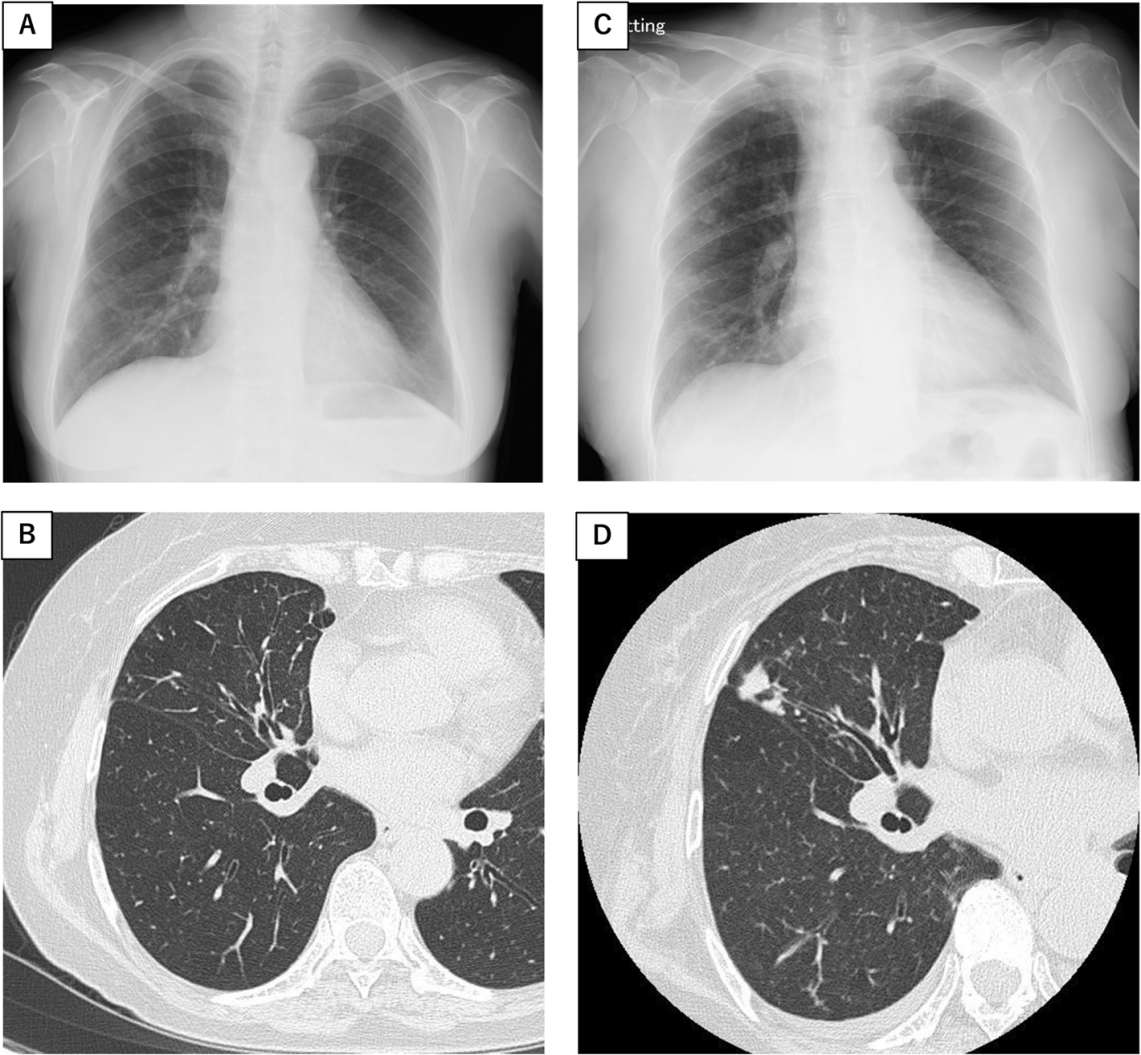
Partial progression of nodular shadow and bronchiectatic changes in right middle lung. The chest X-ray (**a**) and the chest computed tomography (**b**) performed when diagnosed with pulmonary MAC infection (2 years prior to current admission). The chest X-ray (**c**) and the chest computed tomography (**d**) performed at current admission

Upon admission, a physical examination revealed a body temperature of 38.0 °C, a regular pulse rate of 81 beats/min, a respiratory rate of 12 breaths/min, SpO2 of 99% (room air), and a blood pressure of 163/86 mmHg. With the exception of pitting edema of the lower leg, the findings of the physical examination were unremarkable. Blood analysis revealed the following: white blood cells, 8440/μL; hemoglobin, 9.8 g/dL; platelets, 27.4/μL; blood urea nitrogen, 92.0 mg/dL; sCr, 10.85 mg/dL; C-reactive protein (CRP), 16.64 mg/dL; albumin, 2.5 g/dL; MPO-ANCA, 94.1 U/mL (normal < 3.0 U/mL); proteinase 3 (PR3)-ANCA, < 3.5 U/mL (normal < 3.5 U/mL); and anti-GBM antibody, 613 U/mL (normal < 7 U/mL). Urine test results were as follows: protein, 2+; daily urinary protein excretion, 2.36 g/gCr; occult blood, 3+; urinary sediment of red blood cells, > 100 /μL; urinary N-acetyl-β-D-glucosaminidase (NAG), 10.6 U/mL, and urinary β2-microglobulin, 30,334 μg/L. Although chest computed tomography (CT scan) did not indicate alveolar hemorrhage, the partial progression of a nodular shadow with bronchiectatic changes in the right middle lung was observed compared with a CT scan from 2 years prior (Fig. 1). This was assumed to be a lesion due to the MAC infection.

**Fig. 2:**
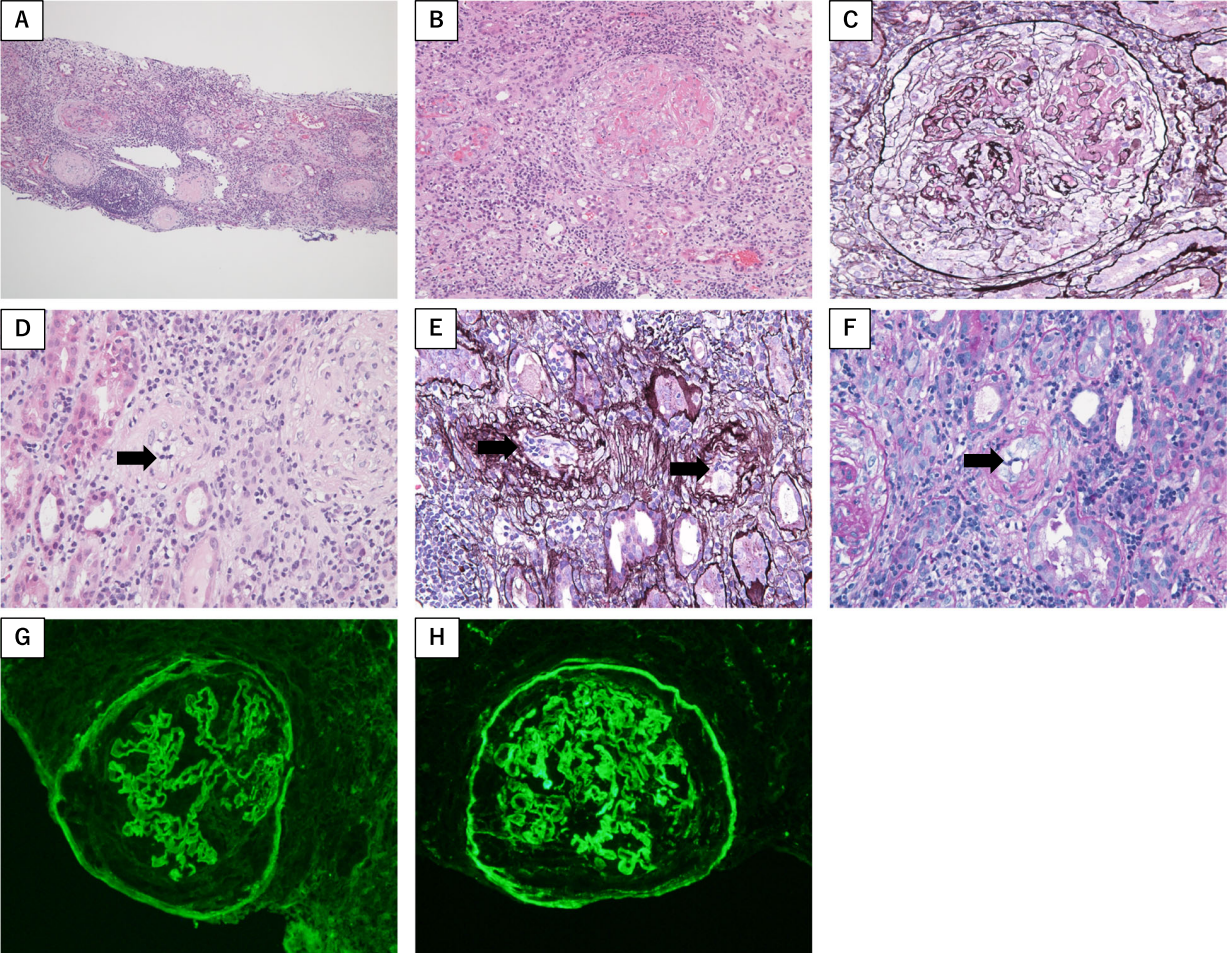
Pathological findings of the renal biopsy in the present case. Kidney specimens were stained with hematoxylin and eosin stain (**a**: low power field; **b**, **d**: high power field), periodic acid methenamine silver (**c**, **e**: high power field) and periodic acid-Schiff (PAS) stain (**f**, **g**: high power field). Light microscopy showed global sclerosis, tubulitis, interstitial inflammation (**a**), cellular crescents with segmental fibrinoid necrosis (**b**, **c**), and endarteritis of arterioles showing the accumulation of mononuclear cells in the arteriole walls (indicated by arrows) (**d**, **e**, **f**). Immunofluorescence microscopy showed linear deposits of IgG (**f**) and C3c (**g**) along the glomerular basement membrane (GBM)

A renal biopsy was performed the day after admission and analyzed using light and immunofluorescence microscopy (Fig. 2). Twenty-three glomeruli were observed, eleven of which showed global sclerosis (Fig. 2a), and ten of the remaining twelve showed cellular crescents with segmental fibrinoid necrosis (Fig. 2b and c). Interstitial lesions in the kidney were characterized by tubulitis and interstitial inflammation (Fig. 2a), and endarteritis of arterioles (Fig. 2d, e, f). Immunofluorescence microscopy showed linear deposits of immunoglobulin G (IgG) and complement 3c (C3c) along the GBM (Fig. 2f and g, respectively). Therefore, RPGN could have been induced by both anti-GBM nephritis and AAV.

**Fig. 3:**
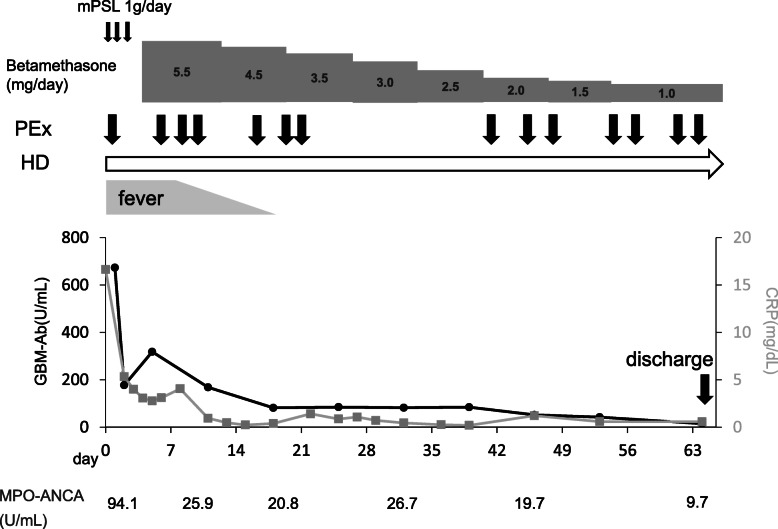
Clinical course of present case. CRP: C-reactive protein; mPSL: methyl-prednisolone; GBM-Ab: anti-GBM antibody; HD: hemodialysis; PEx: plasma exchange

The clinical course of the patient from day of admission through post-discharge was documented in detail (Fig. 3). Steroid pulse therapy [methylprednisolone (1000 mg, daily for three consecutive days)] and plasma exchange were initiated on the day of admission, followed by oral betamethasone (0.1 mg/kg/day) and hemodialysis. On day 65, the anti-GBM antibody titer, the MPO-ANCA titer, and serum CRP levels decreased to 15.0 U/mL, 8.7 U/mL, and 0.57 mg/dL, respectively. However, renal function did not recover and the patient remained on hemodialysis. No other lesion of vasculitis, for example an alveolar hemorrhage, was detected. The pulmonary NTM infection did not progress, and no anti-tuberculosis therapy was administered. Betamethasone was tapered to 1.0 mg/day, and the patient was discharged on day 69. Hemodialysis was continued after discharge.

## Discussion and conclusions

The pathological finding of renal lesions in this case demonstrated not only the features of anti-GBM nephritis such as linear deposits of IgG and C3c along the GBM, but also those of AAV including endarteritis of interlobular artery. Based on these pathological findings, this case was diagnosed with both anti-GBM nephritis and AAV.

Patients with anti-GBM disease, also known as Goodpasture’s disease, often develop both RPGN and pulmonary hemorrhage; however, 30–40% have renal involvement alone [7]. The suspicion of anti-GBM disease can usually be easily validated by the detection of serum anti-GBM antibodies using either indirect immunofluorescence or direct enzyme-linked immunoassay; however, a definitive diagnosis is made by means of a renal biopsy. Optical microscopic findings in renal biopsies consistent with anti-GBM disease usually include crescentic glomerulonephritis, whereas immunofluorescence microscopy demonstrates the virtually pathognomonic finding of linear deposits of IgG along the GBM.

It has been suggested that both environmental factors such as lung infections, exposure to hydrocarbons, and smoking, combined with genetic factors may cause anti-GBM disease [8, 9]. Recent reports suggested the association between pulmonary tuberculosis infection and anti-GBM disease [10, 11]. These risk factors are believed to expose a cryptic epitope in the alveolar basement membrane thereby inducing an autoimmune response against the α3 chain of type IV collagen and the subsequent anti-GBM disease [12].

Compared with anti-GBM disease, pathological renal findings for AAV are characterized as necrotizing vasculitis predominantly in small vessels with few or no immune deposits and is associated with ANCAs specific for MPO or PR3 [13].

Several reports have described a role for ANCAs in the mechanism of GBM antibody production [2, 3]. ANCA has a strong membrane-disordering activity. An analysis of longitudinal sera collected prior to the onset of GBM nephritis indicated that as many as 40% of GBM nephritis patients have ANCA before anti-GBM antibodies are detected [14]. Thus, the presence of ANCA has been speculated to be partly responsible for the development of anti-GBM nephritis. Importantly, AAV exposes the NC1 domain of the α3 chain of type IV collagen in lesions of glomerular or alveolar basement membrane, which could result in the production of anti-GBM antibodies.

In this case study, it was not clear whether the glomerular lesion was caused by AAV because the renal biopsy revealed linear deposits of IgG and C3c predominantly along the GBM, which is evidence of anti-GBM nephritis rather than AAV. However, also in this case, the interstitial lesion was characterized as endarteritis of arterioles. Arterioles are considered to be one of the target vessels of AAV [13], and endarteritis of arterioles are not caused by anti-GBM nephritis because type IV collagen is not a constituent of an arteriole. Therefore, we suggest that both AAV and anti-GBM nephritis are responsible for the renal lesions in this patient.

Following the clinical course of the patient, serum ANCA were detectable before the onset of renal lesions, and anti-GBM antibodies became detectable later at the onset of GBM nephritis, suggesting that ANCA may have induced the development of anti-GBM antibodies in this patient similar to the previously cited reports. Taken together, both ANCAs and anti-GBM antibodies could have played an important role in the pathogenesis of renal lesions in this case.

In several studies, respiratory tract infections, including NTM or other infections, were associated with the development and increase of serum ANCA [15]. Recently, an increase in the occurrence of NTM has been reported in Japan [16], and MAC is considered to be the cause for most pulmonary NTM infections. It has been suggested that NTM infection is related to the development of AAV [5, 6]; however, the mechanism by which NTM induces ANCA production is not fully clarified.

Conversely, the mechanism of ANCA induction by *Mycobacterium tuberculosis*, which belongs to the genus mycobacterium along with NTM, is well known. Thus, M. tuberculosis stimulates the release of oxygen metabolites from neutrophils that are activated through interactions with the phenol glycolipids in the M. tuberculosis cell wall [17]. This activation most likely leads to the release of lysosomal enzymes from the neutrophils in the initial stages of the mycobacterial infection, and autoantibodies against the granular components of the infected cells are produced [18]. Thus, the mechanism by which ANCA production is induced by NTM infection may be similar to that induced by M. tuberculosis. As in this case, NTM infection may be involved in the pathogenesis of some cases of AAV. Therefore, it may be important to measure ANCA in NTM patients with abnormal urine findings.

In this case, histological findings of the renal biopsy showed no remaining normal glomeruli; 11 of 23 were fully sclerotic, 10 were cellular crescentic, and 2 were collapsed glomeruli. Renal biopsy findings showed few normal glomeruli and the renal lesions were determined to be those induced by anti-GBM antibodies rather than those of the AAV. At the time of diagnosis of GBM by the renal biopsy findings, it was considered that immunosuppressive therapy was not expected to improve renal function. Additionally, there was no organ manifestation of AAV or GBM disease other than renal lesions in this patient. In addition, rituximab had not been approved for GBM disease in Japan, therefore the Japanese guideline of RPGN did not recommend rituximab for GBM disease [19].

This patient was treated with multidisciplinary therapy which, unfortunately, did not improve the renal prognosis. The presence of both AAV and GBM nephritis in the renal lesions could be the reason. Although AAV was diagnosed relatively early as a cause of kidney damage, the diagnosis of GBM nephritis was delayed in this case. An early diagnosis of GBM nephritis complicated with AAV might have improved the renal prognosis. It is important to recognize that some cases of AAV are complicated by GBM nephritis, and this could have a strong impact on the renal prognosis as was seen in this case. The detection of anti-GBM antibodies at the time of diagnosis and during treatment of AAV with severe renal impairment would be beneficial in improving renal outcome.

## Data Availability

Not applicable.

## Abbreviations

ANCA: Antineutrophil cytoplasmic antibodies
GBM: Glomerular basement membrane
RPGN: Rapidly progressive glomerulonephritis
NTM: Nontuberculous mycobacteriosis
MPO: Myeloperoxidase
AAV: ANCA-associated vasculitis
MAC: *Mycobacterium avium* complex
PAN: Polyarteritis nodosa
PSL: Prednisolone
CRP: C-reactive protein
PR3: Proteinase 3
CT: Computed tomography
IgG: Immunoglobulin G
C3c: Complement 3c
